# Cómo seleccionar una metasíntesis cualitativa para la práctica basada en la evidencia en salud

**DOI:** 10.15446/rsap.V27n5.122590

**Published:** 2025-09-01

**Authors:** Luz A. Suarez-Jaramillo, Diana P. Betancurth-Loaiza, Francisco J. Tamayo-Giraldo, Brandon Morales-Osorio

**Affiliations:** 1 LS: Enf. Esp. Administración en Salud. M. Sc. Salud Pública. Unidad Central del Valle del Cauca (Uceva), Facultad de Ciencias de la Salud. Tuluá, Colombia. lsuarez@uceva.edu.co Unidad Central del Valle del Cauca Facultad de Ciencias de la Salud Tuluá Colombia lsuarez@uceva.edu.co; 2 DB: Enf. M. Sc. Universitario en Investigación en Atención Primaria. Esp. Administración en Salud. Esp. Promoción de la Salud. M. Sc. Enfermería con énfasis en salud familiar. Ph. D. Salud Pública. Facultad de Ciencias para la Salud, Universidad de Caldas. Manizales, Colombia. diana.betancurth@ucaldas.edu.co Universidad de Caldas Facultad de Ciencias para la Salud Universidad de Caldas Manizales Colombia diana.betancurth@ucaldas.edu.co; 3 FT: MD. Esp. Geriatría. Esp. Medicina Internista. Esp. Gerencia en Salud del Adulto Mayor. M. Sc. Salud Pública. Pontificia Universidad Javeriana de Cali. Santiago de Cali, Colombia. franciscoj.tamayo@javerianacali.edu.co Universidad Javeriana de Cali Santiago de Cali Colombia franciscoj.tamayo@javerianacali.edu.co; 4 BM: Lic. Educación Física, Recreación y Deporte. M. Sc. Actividad Física para la Salud. Facultad de Ciencias para la Salud, Universidad de Caldas. Manizales, Colombia. brandon.morales@ucaldas.edu.co Universidad de Caldas Universidad de Caldas Manizales Colombia brandon.morales@ucaldas.edu.co

**Keywords:** Investigación cualitativa, salud humana, literatura de revisión, revisión sistemática, práctica basada en la evidencia *(fuente: DeCS, BIREME)*, Qualitative research, human health, review literature, systematic review, evidence-based practice *(source: MeSH, NLM)*

## Abstract

La metasíntesis cualitativa permite interpretar y sintetizar conocimientos para comprender fenómenos, informar la práctica clínica, asesorar políticas y desarrollar teoría. De esta forma, se consolida como una estrategia clave para superar enfoques reduccionistas que distorsionan la realidad.

**Objetivo:**

Identificar los aportes metodológicos y prácticos de la metasíntesis cualitativa a la práctica basada en la evidencia.

**Método:**

Se realizó una revisión narrativa. La búsqueda abarcó PubMed, Web of Science, Scopus, Google Académico y Connected Papers.

**Resultados:**

Se incluyeron 30 artículos en los que se identificaron seis tipos de meta-síntesis: metaetnografía, metaestudio, metasíntesis, síntesis interpretativa crítica, síntesis narrativa y meta-síntesis cualitativa.

**Conclusión:**

La metasíntesis es un método potente en la práctica basada en la evidencia, permite interpretar críticamente los estudios cualitativos. Cada enfoque responde a distintas necesidades: la metaetnografía y la metasíntesis cualitativa generan nuevas interpretaciones, mientras que el metaestudio y la síntesis interpretativa crítica ofrecen análisis rigurosos. La selección del método adecuado depende del fenómeno de estudio.

## Abstract (en)

Qualitative meta-synthesis allows for the interpretation and integration of knowledge to understand phenomena, inform clinical practice, guide policy, and develop theory. In this way, it becomes a key strategy to overcome reductionist approaches that distort reality.

**Objective:**

To identify the methodological and practical contributions of qualitative meta-synthesis to evidence-based practice.

**Method:**

A narrative review was conducted. The search covered PubMed, Web of Science, Scopus, Google Scholar, and Connected Papers.

**Results:**

Thirty articles were included, identifying six types of meta-synthesis: meta-ethnography, meta-study, meta-synthesis, critical interpretive synthesis, narrative synthesis, and qualitative meta-synthesis.

**Conclusion:**

Meta-synthesis is a powerful method in Evidence-Based Practice, enabling critical interpretation of qualitative studies. Each approach responds to different needs: meta-ethnography and qualitative meta-synthesis generate new interpretations, while meta-study and critical interpretive synthesis provide rigorous analyses. The choice of the appropriate method depends on the phenomenon under study.

La investigación cualitativa constituye uno de los principales aportes en salud, dado que el conocimiento que genera provoca movimientos y cambios en la formación continuada y en los aspectos prácticos. La investigación cualitativa integra enfoques diversos, pero comparte una epistemología interpretativa que busca comprender cómo las personas interpretan fenómenos y problemas. Esta perspectiva, conocida como hermenéutica, analiza el objeto de estudio en sus partes y como un todo a partir de los significados presentes en los datos 1.

Si la evidencia científica es la forma que ha adoptado inicialmente este movimiento de síntesis y de crítica en las Ciencias de la Salud, el término metasíntesis es el concepto que varios autores han acuñado para referirse a un sistema de conocimiento abierto que pretende la integración de datos, teorías, métodos y cualquier otro tipo de conocimiento desde los hallazgos cualitativos para dar respuesta a problemas complejos 2.

Han surgido diferentes enfoques para las revisiones sistemáticas cualitativas, en 1985 fue utilizado por primera vez por Stern y Harris nombrado como metaanálisis cualitativo en el marco de una teoría explicativa, modelo o descripción 3. La mayoría de los estudios se han relacionado con los procedimientos metaetnográficos delineados originalmente con Noblit y Hare 4, de donde han emergido iteraciones y adaptaciones para el análisis de los fenómenos hasta llegar a diseños más crecientes como las síntesis realistas descrita por Ray Pawson y Nick Tilley 5 desde 2018 con utilidad en la salud pública, las políticas públicas y los contextos complejos 6.

La metasíntesis cualitativa es un campo de investigación emergente con contribución potencial a la práctica basada en la evidencia (PBE) en el campo de la salud, a pesar de controversias conceptuales, epistemológicas y metodológicas.

Se evidencia que las revisiones sistemáticas y los metaanálisis son relativamente conocidos. Sin embargo, la investigación cualitativa ha ido creciendo, encaminándose hacia la problemática de la acumulación de información. Los críticos del campo de la investigación cualitativa consideran que existe un "interruptus analítico" dado que los estudios individuales proporcionan descripciones y perspectivas valiosas, con ausencia de la falta de conexiones establecidas o relaciones horizontales entre los estudios, lo que limita su utilidad 6.

La metasíntesis suele ser confundida con otro tipo de revisiones como las integrativas, las documentales o las narrativas. No obstante, más que un proceso descriptivo, las metasíntesis cualitativas no pretenden simplemente resumir todos los datos disponibles; más bien, presentan nuevas perspectivas sobre temas a través de la interpretación o reinterpretación de los hallazgos con diferentes estudios cualitativos, para crear hallazgos de "tercer nivel" para el avance tanto del conocimiento como de la teoría, mientras que otras metasíntesis no especifican el tipo de diseño 7-9, incluso en las indicaciones para autores de diferentes revistas omiten especificar una ruta clara de desarrollo metodológico.

En suma, la metasíntesis cualitativa es un diseño que permite interpretar y sintetizar la evidencia, de modo que facilita comprender un fenómeno, orientar la práctica, asesorar políticas y desarrollar teoría. Por ello, representa una vía renovada para superar posturas reduccionistas que distorsionan la realidad. El propósito de esta revisión fue identificar los aportes metodológicos y prácticos que ofrece la metasíntesis cualitativa a la práctica basada en la evidencia en salud, con el fin de ayudar al investigador a tomar las mejores decisiones para su estudio.

## MÉTODO

Se realizó una revisión narrativa 10 para contribuir a la PBE en salud desde el abordaje cualitativo. Para lograr el objetivo, se llevaron a cabo los siguientes pasos: búsqueda bibliográfica, selección de los documentos e interpretación de los hallazgos.

### Búsqueda bibliográfica

La búsqueda se realizó en las bases de datos PubMed, Web of Science y Scopus, así como en los motores de búsqueda Google Académico y Connected Papers. Se usaron los términos "investigación cualitativa", "revisión sistemática", "salud" y el término libre "metasíntesis", en español e inglés, combinados con AND: (investigación cualitativa AND revisión sistemática AND salud humana), (qualitative research AND systematic review AND human health) y (qualitative research AND metasynthesis AND human health).

También se consultaron otras fuentes, como las referencias seleccionadas de la bibliografía de los artículos incluidos previamente, así como búsqueda manual y literatura gris.

Los criterios de inclusión fueron artículos científicos, emblemáticos y literatura gris, como libros relacionados con el área de interés, disponibles en texto completo, con metodología cualitativa tipo revisión sistemática, en idioma español, portugués e inglés, enfocados en la interpretación y la síntesis de conocimientos del campo de la salud. Frente a los criterios de exclusión se encuentran artículos de reflexión, artículos con abordaje cuantitativo o mixto, cartas al editor y tesis de grado.

### Selección de los documentos

**Figura 1:**
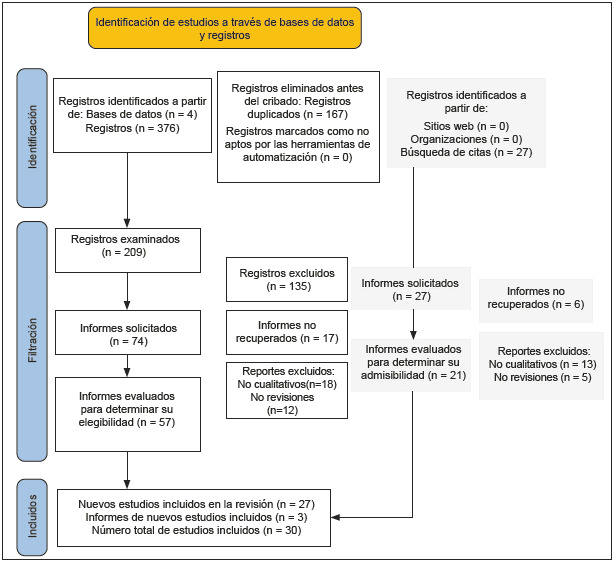
Flujograma PRISMA

Se realizó un proceso de selección en el que se evaluó la idoneidad de los estudios utilizando la metodología Preferred Reporting Items for Systematic Reviews and Meta-Analyses (Prisma) (Figura 1).

Tras ejecutar las ecuaciones de búsqueda, se recuperaron 376 referencias de las bases de datos. La evaluación posterior, mediante título, resumen y revisión del texto completo, permitió la inclusión de 27 referencias que cumplían con los criterios de inclusión establecidos. Además, se integraron tres referencias complementarias mediante una revisión de las listas de referencias y una búsqueda específica de literatura gris, lo que resultó en un conjunto final de 30 documentos.

La selección se realizó mediante la interpretación, la síntesis y el análisis crítico de los artículos seleccionados, en una matriz de donde se extrajo la información relevante de cada estudio con las siguientes categorías: tipo de metasíntesis, abordaje epistemológico, autores relevantes, características relevantes, fases metodológicas y usos en salud de las revisiones, criterios de calidad e instrumentos. El análisis fue realizado conjuntamente por cuatro investigadores (tres estudiantes de doctorado y una doctora en salud pública). Para verificar la calidad de esta revisión se aplicó la escala SANRA (Scale for the Quality Assessment of Narrative Review Articles).

## RESULTADOS

### Tipos de revisiones sistemáticas cualitativas

El Cuadro 1 presenta una categorización de los principales enfoques de metasíntesis cualitativa, diferenciándolos según su abordaje epistemológico, autores representativos, características metodológicas y fases de análisis. Se identifican seis tipos: metaetnografía, que traduce conceptos entre estudios 4; metaestudio, que examina datos, métodos y teoría 2; metasíntesis, que distingue entre metarresumen y síntesis interpretativa 11; síntesis interpretativa crítica, con un enfoque iterativo y evaluativo 12; síntesis narrativa, que integra estudios cualitativos y cuantitativos 13; y metasíntesis cualitativa, orientada a la generación de nuevas interpretaciones 14. Estos enfoques permiten una comprensión profunda de fenómenos complejos, lo cual favorece el desarrollo de teoría y la aplicación del conocimiento cualitativo en distintos campos (Cuadro 1).

**Cuadro 1:** Tipos de metasíntesis identificados en la literatura

Tipo de metasíntesis / abordaje epistemológico / autores	Características relevantes	Fases metodológicas
Meta-etnografía Interpretativo George W. Noblit y R. Dwight Hare 4.	Método para sintetizar estudios cualitativos mediante la “traducción” de conceptos y metáforas de un estudio a otro, buscando generar interpretaciones que vayan más allá de los hallazgos individuales. Obra: Meta-etnografía: sintetizando estudios cualitativos 4.	Identificación del área de interés Selección de estudios relevantes Lectura repetida de los estudios seleccionados Determinación de las relaciones entre los estudios Traducción de los estudios entre sí Síntesis de las traducciones Expresión de la síntesis La síntesis resultante puede adoptar tres formas: traducción recíproca, traducción refutadora, explorar contradicciones entre estudios, línea argumentativa
Meta-etnografía Interpretativo George W. Noblit y R. Dwight Hare 4.	Método integral y flexible para la síntesis del conocimiento que permite desarrollar teorías de rango medio con aplicaciones prácticas. Proporciona un marco estructurado para el muestreo, la evaluación y la síntesis. Su eficacia depende del rigor de los métodos subyacentes. Obra: Meta-study of qualitative health research 2.	Metadatos: síntesis de los datos presentadosen los informes Metamétodo: examina los métodos aplicados en un área de estudio Metateoría: análisis crítico de los marcos teóricos que han orientado la investigación
Metasíntesis Metarresumen (metasumario) Posmodernista y constructivista Margarete Sandelowski & Julie Barroso 15.	Método sistemático, riguroso y reflexivo que trasciende la mera agregación de hallazgos, al reinterpretarlos, integrarlos y generar nuevos marcos teóricos o comprensiones sobre un fenómeno. Privilegia la inclusión de estudios, que permite una representación más integral de la evidencia. La metodología se articula en dos fases basadas en la tipología de los hallazgos: la fase de metarresumen (o metasumario), destinada a la síntesis de hallazgos y patrones en estudios descriptivos, y la fase de metasíntesis, orientada a la interpretación profunda de los hallazgos cualitativos. Obra: Handbook for synthesizing qualitative research 15.	Posicionamiento filosófico: propósito Búsqueda de literatura Evaluación de calidad Clasificación de hallazgos: identificación de unidades significativas de datos cualitativos Síntesis de hallazgos Metarresumen (si se aplica). (a) Extracción de las conclusiones pertinentes de cada informe. Reducción de estas afirmaciones a conclusiones Cálculo del tamaño de los efectos. Esta síntesis: puede usar análisis taxonómico, comparación constante y recíproca, conceptos importados, o traducción y síntesis recíproca. Presentación de resultados: Explicación de patrones emergentes y contribución a la teoría cualitativa.
Síntesis interpretativa crítica Crítico-interpretativo Mary Dixon-Woods, Debbie Cavers, Shona Agarwal, Ellen Annandale, Antony Arthur, Janet Harvey, Ron Hsu, Savita Katbamna, Richard Olsen, Lucy Smith, Richard Riley y Alex J. Sutton 12.	Método desarrollado para abordar las limitaciones de las sistemáticas tradicionales al analizar temas complejos. Permite una evaluación crítica de la literatura, considerando tanto la calidad metodológica como las suposiciones subyacentes de los estudios incluidos. A través de un proceso iterativo, se generan nuevas interpretaciones y teorías que ofrecen una comprensión más profunda del fenómeno estudiado. Obra: Conducting a critical interpretive synthesis of the literature on access to healthcare by vulnerable groups 12.	Formulación de la pregunta de Investigación Búsqueda de la literatura Selección y evaluación crítica de estudios Extracción y análisis de datos Síntesis interpretativa: “traducción recíproca” Desarrollo de teoría
Síntesis narrativa Realista y pragmático Jennie Popay, Helen Roberts, Andrew Sowden, Mark Petticrew, Lisa Arai, Miriam Rodgers, Sandy Duffy 13.	Método que busca interpretar y resumir hallazgos de estudios diversos, tanto cualitativos como cuantitativos, especialmente cuando no es apropiado o posible realizar un metaanálisis cuantitativo. Permite integrar evidencias de estudios con diferentes diseños y metodologías, a través del análisis de resultados en diferentes estudios de una manera profunda y sistemática, proporcionando una comprensión más completa de un fenómeno. Obra: Guidance on the conduct of narrative synthesis in systematic reviews: A product from the ESRC Methods Programme Book 13.	Desarrollo de una teoría inicial Desarrollo de una síntesis inicial Exploración de las relaciones en los datos Evaluación de la solidez de la síntesis
Metasíntesis cualitativa. Pragmático e interpretativo Deborah Finfgeld-Connett 14.	Método para integrar y reinterpretar hallazgos de estudios cualitativos previamente publicados. Busca generar un conocimiento más profundo y teóricamente enriquecido a partir de múltiples fuentes de datos cualitativos. Obra: A guide to qualitative meta-synthesis 14.	Formulación de la pregunta de investigación Búsqueda exhaustiva de literatura relevante Evaluación de la calidad de los estudios seleccionados Extracción y análisis de datos Síntesis de los hallazgos para generar nuevas interpretaciones

### Tronco común entre metasíntesis

**Figura 2:**
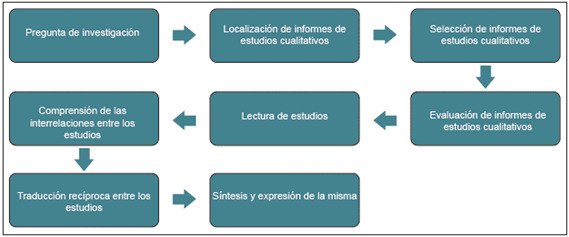
Fases generales de una meta-síntesis

Las metasíntesis siguen estas fases generales: formular la pregunta, localizar y filtrar los estudios cualitativos, evaluarlos, leerlos a fondo, identificar sus interrelaciones, generar traducciones recíprocas (entendida como un proceso de comparación e intercambio constante entre diferentes tipos de información o datos para analizar similitudes y diferencias), y, finalmente, sintetizar los hallazgos para su informe (véase Figura 2).

La mayoría de los documentos detallan sus estrategias de búsqueda sistemática (bases de datos, términos de búsqueda, fechas, entre otros). El cribado y la selección son rigurosos, a menudo con doble revisión y resolución de discrepancias. La extracción de datos generalmente implica la identificación de temas, categorías o conceptos de primer y segundo orden. El análisis y la síntesis se describen a través de técnicas como el análisis temático, la comparación constante, la agrupación de códigos, la construcción de metáforas o la traducción recíproca. La reflexión del equipo y la coconstrucción del significado son prácticas comunes para asegurar el rigor.

### Criterios de calidad

Las metasíntesis revisadas emplean distintos criterios para valorar la calidad de los estudios cualitativos seleccionados 16, como se indica en el Cuadro 2.

**Cuadro 2:** Criterios generales para la valoración de estudios cualitativos primarios e informe de la metasíntesis

Criterios generales para la selección de artículos	Instrumentos que evalúan la calidad de los estudios cualitativos primarios	Guía de presentación de informe y valoración de la calidad de la metasíntesis
Relevancia conceptual y teórica Congruencia metodológica Exploración de la heterogeneidad del fenómeno Credibilidad de los hallazgos, fieles a la experiencia de los participantes Reflexividad del investigador Transparencia en el reporte	Consolidated criteria for reporting qualitative research (COREQ) CASP (Critical Appraisal Skills Programme) JBI Critical Appraisal Checklist for Qualitative Research (Joanna Briggs Institute) The Qualitative Assessment and Review Instrument (QARI) Primary Research Appraisal Tool- Q (PRAT-Q) MMAT (Mixed Methods Appraisal Tool	Preferred Reporting Items for Systematic Reviews and Meta-Analyses Prisma Enhancing transparency in reporting the synthesis of qualitative research: ENTREQ Improving reporting of meta-ethnography: the eMERGe reporting guidance

El uso del Critical Appraisal Skills Programme (CASP) y las directrices Prisma se citaron ampliamente para la transparencia en el reporte de los procesos de revisión. En algunos casos, las revisiones que adoptaron el tipo de síntesis interpretativa crítica no utilizaron un enfoque formal de evaluación de calidad, justificado en el interés conceptual y la heterogeneidad de las fuentes, basándose en un "juicio subjetivo", en lugar de criterios metodológicos. Solo tres revisiones utilizaron checklists formales como ENTREQ o GRADE-CERQual de manera explícita, sin embargo, muchos estudios describen cómo abordaron la confianza de los hallazgos, a saber: reflexividad, triangulación, participación de múltiples revisores o bibliote-cólogos para la búsqueda de los artículos.

### Análisis de categorías

En las metasíntesis analizadas, no se identifica un tipo de análisis concreto, aunque la teoría fundamentada es el enfoque más recurrente, mientras que Dixon-Woods 12 en la síntesis interpretativa crítica menciona explícitamente el análisis marco. La teoría fundamentada, desarrollada por Glaser y Strauss 17 constituye un método central en el análisis cualitativo, basado en el interaccionismo simbólico para interpretar los significados emergentes en las interacciones sociales. Su proceso implica una codificación iterativa mediante comparación constante, lo que permite generar conceptos y categorías teóricas progresivamente más abstractas. Estas categorías presentan propiedades y dimensiones que varían según los participantes y el contexto del estudio. Para garantizar el rigor teórico, la metodología debe ser auditable a través del registro sistemático de notas analíticas y decisiones de codificación, asegurando transparencia y validez 18,19.

Además de la teoría fundamentada, pueden encontrarse otros enfoques analíticos: el análisis temático permite identificar y organizar estructuradamente temas relevantes dentro de los datos 20; el análisis de marco facilita el procesamiento de grandes volúmenes de información mediante el mapeo de temas, definidos inductiva o deductivamente según los objetivos del estudio 1. Por su parte, el análisis de casos cruzados posibilita la comparación de distintos casos a través de reducción, presentación y formulación de conclusiones, estructurando el análisis de datos heterogéneos 21,22.

### Estudios en el ámbito de la salud que han empleado la metasíntesis

Se destacan ejemplos de metasíntesis cualitativa en salud para demostrar algunas de las contribuciones actuales de este diseño y su utilidad para futuras investigaciones en este campo. Las diferentes metasíntesis en salud permiten analizar fenómenos desde múltiples enfoques. La metaetnografía es ideal para comprender percepciones y prácticas culturales en salud, identificando barreras socioculturales en la atención. El metaestudio abordó la comprensión de experiencias en asuntos psicosociales sensibles en contextos de vulnerabilidad, exclusión o transformación. La metasíntesis integra experiencias de pacientes y profesionales, evaluando la calidad de la atención y la relación entre cuidadores y personas con enfermedades crónicas. La síntesis crítica interpretativa se enfoca en el análisis de sistemas de salud y políticas sanitarias, proporcionando una visión estructural de barreras en el acceso y la comunicación médico-paciente. La síntesis narrativa permite integrar evidencia diversa para evaluar el impacto organizacional y sistémico en la atención sanitaria, incluyendo el bienestar de los profesionales de la salud. Finalmente, la metasíntesis cualitativa profundiza en las experiencias de poblaciones vulnerables, identificando barreras y facilitadores en el acceso a los servicios de salud. En conjunto, estas estrategias contribuyen a la mejora de la atención sanitaria desde una perspectiva cualitativa y basada en evidencia (Cuadro 3).

**Cuadro 3:** Ejemplos de estudios en el ámbito de la salud que han empleado la metasíntesis

Ejemplo del uso de metasíntesis en salud (Título del estudio/ autores/DOI)	Fenómeno / población
Tipo de metasíntesis/autor metaetnografía/Noblit & Hare 4	
Facilitators and barriers to seeking and engaging with antenatal care in high-income countries: A meta-synthesis of qualitative research. Escañuela 23 https://doi.org/10.1111/hsc.14072. COVID-19 and nurse faculty caring: A meta-synthesis. Ntshingila 24https://doi.org/10.1016/j.heliyon.2024.e28472. What shapes local health system actors’ thinking and action on social inequalities in health? A meta-ethnography. McMahon 25 Breastfeeding in Indigenous communities: A meta-ethnography of knowledge and practices. Pico 26https://search.informit.org/doi/epdf/10.3316/informit.T2024032200014200483814322. Understanding ethnic inequalities in mental healthcare in the UK: A meta-ethnography. Bansal 27https://doi.org/10.1371/journal.pmed.1004139.	Atención prenatal/mujeres gestantes Atención a pacientes/estudiantes de enfermería Desigualdades en salud/agentes del sistema sanitario Lactancia materna exclusiva/mujeres indígenas Desigualdades étnicas/grupos étnicos minoritarios
Tipo de metasíntesis / Autor: Metaestudio / Paterson 2	
Women’s experiences with yoga after a cancer diagnosis: a qualitative meta-synthesis-part I. Price 28 https://doi.org/10.1186/s13643-023-02350-x. Care and support when a baby is stillborn: A systematic review and an interpretive meta-synthesis of qualitative studies in high-income countries. Persson 29 https://doi.org/10.1371/journal.pone.0289617. Children’s knowledge about play-related risk, risk-taking, and injury: a meta-study. McCallum 30 https://doi.org/10.1080/14927713.2023.2242859. Identity in elite level disability sport: a systematic review and meta-study of qualitative research. Crossen 31 https://doi.org/10.1080/1750984X.2023.2214993. Identidad en el deporte/personas con discapacidad. Stroke experiences and unmet needs of individuals of African descent living in high-income economy countries: a qualitative meta-synthesis. Singh 32 https://doi.org/10.1007/s40615-023-01725-z.	Yoga y cáncer/mujeres. Muerte fetal/padres y profesionales de la salud. Riesgo en el juego/niños entre 5 y 16 años. Identidad en el deporte/personas con discapacidad. Necesidades culturales e individuales/pacientes con ictus.
Tipo de meta síntesis/Autor: Meta-sintesis/ Sandelowski & Barroso 15.	
Experiences of pain communication in endometriosis: A meta-synthesis. Robstad 33https://doi.org/10.1111/aogs.14995. From qualitative meta-summary to qualitative meta-synthesis: introducing a new situation-specific theory of barriers and facilitators for self-care in patients with heart failure. Herber 34 https://doi.org/10.1177/1049732318800290. Dyadic relationships between informal caregivers and older adults with chronic heart failure: a systematic review and meta-synthesis. Yoong 35 https://doi.org/10.1093/eurjcn/zvae093. core dimensions of social inclusion for residents with mental health and/or substance use challenges: a qualitative meta-synthesis. Ogundipe 36 https://doi.org/10.1007/s40737-024-00415-1. Patients’ experiences of delirium: A systematic review and meta-summary of qualitative research. Kuusisto◻Gussmann 37 https://doi.org/10.1111/jan.14865.	Prestación de servicios de atención sanitaria/pacientes con endometriosis. Barreras y facilitadores de autocuidado/pacientes con falla cardiaca. Relaciones diádicas/cuidadores informales y adultos mayores. Inclusión social/personas con problemas de salud mental y/o consumo de sustancias. Experiencia de delirio/hombres y mujeres hospitalizados.
Tipo de metasíntesis /autor: Síntesis interpretativa crítica / Dixon-Woods (12)		
La comunicación hospitalaria entre profesionales de la salud y familiares de pacientes hospitalizados: Una revisión integradora. López 38 https://doi.org/10.46377/dilemas.v9i2.3146. Domains and processes for institutionalizing evidence-informed health policy-making: a critical interpretive synthesis. Kuchenmüller 39 https://doi.org/10.1186/s12961-022-00820-7. Understanding access to general practice through the lens of Candidacy: a critical review of the literature Sinnott 40 https://doi.org/10.3399/BJGP.2024.0033. Body image concerns in individuals diagnosed with benign gynecological conditions: scoping review and meta synthesis Sayer-Jones 41 https://doi.org/10.1080/21642850.2021.1920949. Candidacy 2.0 (CC) - an enhanced theory of access to healthcare for chronic conditions: lessons from a critical interpretive synthesis on access to rheumatoid arthritis care. Koehn 42https://doi.org/10.1186/s12913-024-11438-6.	La comunicación hospitalaria/profesionales de la salud y familiares de pacientes hospitalizados. Formulación de políticas basadas en la evidencia/sistema de salud. Acceso a candidatura general para la práctica en salud/candidatos a prácticas generales en salud. Condiciones ginecológicas benignas/mujeres en edad reproductiva. Acceso al cuidado de la artritis reumatoidea/pacientes con artritis reumatoidea.
Tipo de metasíntesis /Autor: Síntesis narrativa / Popay 13	
Psychological therapists’ experiences of burnout: A qualitative systematic review and meta-synthesis. Vivolo 43 https://doi.org/10.1016/j.mhp.2022.200253. Risk Factors for pressure injuries in adult patients: a narrative synthesis. Chung 44https://doi.org/10.3390/ijerph19020761. A systematic review of suicidal behaviour in men: A narrative synthesis of risk factors. Richardson 45 https://doi.org/10.1016/j.socscimed.2021.113831. The contribution of political skill to the implementation of health services change: a systematic review and narrative synthesis. Clarke 46 https://doi.org/10.1186/s12913-021-06272-z. The contribution of organisational factors to vicarious trauma in mental health professionals: a systematic review and narrative synthesis. Sutton 47 https://doi.org/10.1080/20008198.2021.2022278.	Fenómeno de Burnout/terapeutas en psicología. Seguridad del paciente/adultos. Comportamiento suicida/hombres. Herramientas para hacer cambios en los servicios de salud/servicios de salud. Papel de factores organizacionales en la disminución/profesionales en general.
Tipo de metasíntesis/ Autor Metasíntesis cualitativa/ Finfgeld-Connett 14	
Interacción entre profesionales de la salud y padres de niños o adolescentes con epilepsia: una revisión sistemática cualitativa y meta-síntesis. Lee 48 https://doi.org/10.1016/j.yebeh.2024.109940. Barreras y facilitadores del acceso a la asistencia sanitaria entre inmigrantes con discapacidad: una meta síntesis cualitativa. Ngondwe 49. https://doi.org/10.3390/healthcare13030313. El trabajo de la lactancia materna entre mujeres de bajo nivel socioeconómico: una meta síntesis cualitativa. Weston 50 https://doi.org/10.1177/23333936231161130. El acceso de las mujeres inmigrantes a los servicios de salud en los Estados Unidos: una metaanálisis cualitativo. Melaku 51 https://doi.org/10.1080/01488376.2022.2035300. El estigma que sufren las mujeres perinatales con dependencia de opioides en los Estados Unidos: una metasíntesis cualitativa. Morton 52 https://doi.org/10.1177/01939459231182495.	Desafíos emocionales y logísticos/padres de niños adolescentes con epilepsia. Barreras, facilitadores y experiencias vividas/inmigrantes con discapacidad. Barreras y facilitadores de la lactancia materna/madres gestantes. Revelar las voces colectivas y experiencias de vida/mujeres inmigrantes. El estigma/mujeres perinatales con dolor y dependencia de opioides.

## DISCUSIÓN

Los resultados de esta revisión evidencian que la meta-síntesis cualitativa es una herramienta clave para integrar hallazgos diversos en salud, dado que brinda síntesis descriptivas, así como teorías e interpretaciones que potencian la transformación práctica. Los seis enfoques identificados: metaetnografía 4, metaestudio 2, metasíntesis 11, síntesis interpretativa crítica 12, síntesis narrativa 13 y metasíntesis cualitativa 14 se caracterizan por una diversidad epistemológica y metodológica que nutre la práctica basada en evidencia desde el paradigma cualitativo.

### Características

Los diferentes métodos de metasíntesis presentan fortalezas y limitaciones según sus niveles de interpretación, rigor metodológico, flexibilidad y su contribución a la generación de teoría 53,54. Desde un análisis comparativo, métodos como la metaetnografía y la síntesis interpretativa crítica destacan por su profundidad teórica e interpretativa, permitiendo la generación de conocimiento innovador a partir de datos previamente publicados. No obstante, esta riqueza teórica exige una rigurosidad metodológica y experticia del investigador, lo cual puede limitar su aplicabilidad en contextos con menor madurez investigativa 55,56.

Por otro lado, el metaestudio se distingue por su rigor metodológico y nivel interpretativo, considera el impacto de los métodos utilizados en los resultados obtenidos, lo que le otorga un rigor epistemológico, convirtiéndolo en una herramienta para la reconstrucción teórica 57. Sin embargo, su flexibilidad es baja y la interpretación introduce un nivel considerable de subjetividad que depende del investigador, limitándose de esta forma su aplicabilidad en diferentes contextos.

Asimismo, el metarresumen y la síntesis narrativa, al privilegiar la flexibilidad metodológica y la inclusión de estudios diversos, permiten aproximaciones útiles para contextos de toma de decisiones rápidas o síntesis preliminares. Debe considerarse que su capacidad para generar teoría es menor y existe el riesgo de simplificación o pérdida de profundidad si no se aplican criterios de rigurosidad 56,58.

La revisión evidencia además que la teoría fundamentada sigue siendo un referente analítico dominante, si bien otros métodos como el análisis temático, el marco analítico o el análisis de casos cruzados emergen como opciones complementarias valiosas. Este pluralismo metodológico demanda una toma de decisiones coherente entre el tipo de metasíntesis, el fenómeno de estudio y los objetivos de la investigación.

### Criterios de calidad

La evaluación de la calidad en las metasíntesis cualitativas es un campo en construcción. A diferencia del paradigma cuantitativo, donde se ha consolidado un marco homogéneo (GRADE), en el ámbito cualitativo prevalece una diversidad epistemológica y enfoques flexibles 12,15.

Autores como Finfgeld-Connett 14 y Paterson 2 coinciden en que los criterios deben adaptarse al propósito de la síntesis. Por ejemplo, mientras el meta-estudio exige un rigor metodológico y atención a la calidad de los métodos, la síntesis interpretativa crítica pone el énfasis en la reflexividad y el cuestionamiento de supuestos, más que en una evaluación estandarizada.

Aunque herramientas como CASP, COREQ o la lista JBI son útiles para estudios primarios, la síntesis requiere marcos propios. ENTREQ 59 y eMERGe 60 aportan lineamientos específicos para reportar con transparencia, mientras que GRADE-CERQual 61 permite valorar la confianza en los hallazgos. Sin embargo, su uso sigue siendo limitado 6, y muchos estudios carecen de justificación explícita de los criterios aplicados.

La revisión de la evidencia demuestra que no hay un solo estándar de calidad, sino que debe ser congruente con el tipo de metasíntesis, el abordaje epistemológico y el nivel de interpretación buscado. La transparencia en las decisiones metodológicas y la reflexividad del equipo investigador son fundamentales para garantizar la credibilidad y la transferibilidad de los hallazgos.

### Estudios de salud

Los aportes a la salud de este tipo de diseños han ido en aumento. Históricamente, la PBE en salud ha sentado las bases como un proceso sistemático y riguroso que orienta la toma de decisiones clínicas basada en los hallazgos de los estudios más robustos de la investigación biomédica 62,63. Sin embargo, el enfoque predominante en la eficacia estadística a menudo omite las complejidades inherentes a la experiencia de los profesionales de la salud 64 y la experiencia humana en el proceso salud-enfermedad, así como los factores contextuales que determinan la aceptabilidad y viabilidad de las intervenciones 65.

Esta limitación es particularmente pronunciada en el ámbito de las intervenciones de salud complejas y las iniciativas de salud pública, dado que los factores sociales, culturales e individuales son de suma importancia. En este sentido, la metasíntesis puede actuar como un puente esencial entre las experiencias individuales y una comprensión teórica amplia de los fenómenos para orientar y complementar decisiones contextualizadas en el ámbito poblacional 66.

Noyes et al. 67 reconocen el potencial de la contribución de la evidencia cualitativa para la toma de decisiones con relación al funcionamiento de intervenciones en salud, al aumentar la comprensión de un fenómeno de interés, la identificación de los vínculos con el contexto, los valores, las actitudes y las experiencias en relación con las condiciones de salud de las personas o los profesionales que intervienen.

Los estudios analizados dejan ver las experiencias vividas por los individuos y los fenómenos complejos en el ámbito de la salud 68. Presentan temas en el continuo de la salud y la enfermedad, desde la prevención y el manejo de condiciones crónicas hasta las experiencias de atención y la formulación de políticas en áreas de salud cardiovascular y enfermedades crónicas, salud de la mujer, salud mental y bienestar social, experiencias de enfermedad y atención médica entre pacientes y personal sanitario, acceso al sistema de salud y política sanitaria.

Un valor significativo de estas metasíntesis es su capacidad para visibilizar las experiencias de poblaciones que enfrentan desventajas y estigma en los sistemas de salud. Se evidencian experiencias y necesidades no cubiertas de individuos de ascendencia africana, barreras sistémicas y personales que enfrentan las mujeres inmigrantes al acceder a la atención médica en los Estados Unidos, incluyendo la discriminación y la falta de competencia cultural, las tensiones entre el conocimiento comunitario y el de los profesionales de la salud sobre lactancia materna en comunidades indígenas, visibilización del estigma de mujeres consumidoras de opioides en su etapa perinatal y cómo esta experiencia puede impedir que las mujeres accedan a la atención, así como desigualdades étnicas en la atención de salud mental.

Existe una diferencia en los documentos, entre aquellos que buscan una comprensión interpretativa profunda y la generación de nuevas teorías, y aquellos que son más descriptivos o agregativos. Para Sim 54, la función de la metasíntesis no es reproducir la riqueza de detalles del mundo empírico que se busca en los estudios primarios, sino analizar interpretaciones más amplias del mundo empírico. En este caso, los estudios que utilizan la metasíntesis cualitativa generadora de teoría 14, la metaetnografía y la síntesis interpretativa crítica son los ejemplos más claros.

La mayoría de los estudios demuestran una clara adherencia a metodologías de síntesis cualitativa establecidas, lo que aumenta su rigor y transparencia 69,70. Pocos estudios usaron las herramientas de ENTREQ o GRADE-CERQual para abordar la confianza de los hallazgos. Se reconoce que la calidad de la metasíntesis no puede ser superior a la calidad de los estudios individuales que la componen.

Los documentos analizados demuestran el creciente rigor y la sofisticación de la síntesis de evidencia cualitativa en la investigación en salud. La variedad de temas y la visibilidad de experiencias de grupos históricamente marginados y violentados son fundamentales para una atención equitativa y centrada en la persona 71. Existe una clara distinción entre las metodologías que aspiran a la generación de teoría y una interpretación abstracta profunda y aquellas que se centran en la descripción y agregación temática. Ambas son valiosas, pero cumplen propósitos distintos: las primeras contribuyen al avance del conocimiento teórico y conceptual, mientras que las segundas sistematizan la evidencia existente para informar directamente la práctica y las políticas. Todos, sin embargo, comparten el objetivo de informar la toma de decisiones clínicas y de salud pública para mejorar la atención y los resultados de salud 67.

En su esencia, la metasíntesis cualitativa es un proceso inductivo invaluable. Tal como lo señalan Kinn et al. 72, va más allá de la simple recopilación: implica un meticuloso comparar, contrastar y traducir las ideas centrales, los conceptos clave y los hallazgos originales de estudios individuales. A pesar de su potencial, la metasíntesis no está exenta de debates y críticas. Persisten inquietudes en algunos sectores de la investigación en ciencias sociales de la salud sobre su utilidad y validez 56.

Finalmente, Los estudios revisados demuestran la aplicabilidad de cada enfoque en temas clave de salud como atención prenatal, salud mental, cuidados paliativos, in-equidades sociales, estigma o experiencias hospitalarias. Esto reafirma que la metasíntesis cualitativa, más que una técnica, es una estrategia crítica de investigación capaz de conectar voces fragmentadas, generar evidencia contextualizada y enriquecer el diseño de intervenciones participativas y situadas.

La metasíntesis como una interpretación de hallazgos cualitativos es clave para comprender fenómenos sociales complejos que afectan la salud. En la investigación sanitaria, es un aporte para avanzar de forma rigurosa en la práctica formal basada en la evidencia de los profesionales de la salud.

Cada tipo de metasíntesis presenta sus propias fortalezas y debilidades, según la profundidad con la que interpretan los hallazgos, el rigor con el que aplican sus métodos, su adaptabilidad y, en última instancia, su capacidad para ayudar a construir nuevas teorías. En esencia, la elección depende del fenómeno de estudio, del objetivo planteado y de la experiencia del investigador en la metodología cualitativa ♣
